# On the Effects of Large Doses of the Sulphate of Quinine

**Published:** 1847-04

**Authors:** Wm. Alexander Thom

**Affiliations:** Eastville, Northampton County, Virginia


*On the Effects of large doses of the Sulphate of Quinine.* By Wm. Alexander Thom, M. D., of Eastville, Northampton County, Virginia.

To the Editor of the Medical Examiner.

In these days of ultraism and exclusiveness in medical matters, I have thought it might not be amiss to report the following cases, illustrative of some of the effects of very large and frequently repeated doses of quinine. I regret that the imperfect notes of the cases preserved, will permit my giving little more than an outline of the symptoms and treatment; enough, however, remains to show that the quinine was the direct cause of the unpleasant symptoms occurring at the conclusion of the treatment; and although all the cases ultimately did well, they still warn us of the dangers attending the indiscriminate administration of this most valuable, but much abused agent. All the cases occurred in the course of the past fall, and three out of four in children of from six to eight years of age, and all were ill with some modification of miasmatic fever, assuming the ordinary remittent or congestive form distinguishing our autumnal diseases. The fourth case will be found to furnish another instance of amaurosis produced by quinine, and affords additional evidence of the correctness of Dr. McLean's opinion on this subject, as expressed in an article transferred to the "Record" of the *Examiner* for February of this year. This was the first case of the kind which had ever come under my observation, in which the effect so immediately followed the administration of the remedy; and I was somewhat at a loss whether to ascribe it to this or not. Dr. McLean's paper, however, has satisfied me on this point.

The phenomena presented by all the cases are explicable only on the idea that quinine exerts a powerfully sedative action on the nervous system. The symptoms manifested,—though in a less degree,—were not unlike those exhibited by some constitutions under the peculiar action of mercury, and described by Mr. Pearson as *mercurial erethism.* Smaller doses of the medicine had, in all the cases, been tried in vain, and I am satisfied that nothing but very large and frequently repeated doses, could have saved the lives of two of them. It will be seen, however, that in most of the cases the quantity of quinine taken was not *inordinately* great.

*Case* 1. A little girl, æt. 7, with remittent of the ordinary form, exacerbation occurring daily about noon. When I saw her, the remission had just begun; I ordered two grains of quinine in solution every hour until she had taken twelve grains; afterwards a cathartic of sulphate of potassa and rhubarb. The next day her fever recurred at the usual time as high as ever. *Pre-*

*scription.*—℞. Hydrarg. chlor. mit. gr. vj., pulv. ipecac. gr. iij. M. Div. in chart. 6.—S. One every two hours; and afterwards, as the paroxysm was expected in four hours, to take five grains of quinine, and repeat it in an hour. She took ten grains; fever rose as usual. At the next remission I ordered her to have sixteen grains of quinine at two doses, with an interval of two hours. When the next paroxysm was expected, I found my patient free from fever, but with a quick, rather weak and thready pulse, mind slow, tongue tremulous, skin cool and moist. All these symptoms subsided entirely in less than twenty-four hours, and she has never had a fever since.

*Case* 2. Negro boy, æt. 6 or 7, ill with severe remittent fever with powerful determination to the brain, accompanied by a high grade of arterial excitement. I bled him, and ordered a solution of nitrate of potassa and tartar emetic, as he had been purged previously to my seeing him. An indistinct remission occurred for a short time every morning; as soon as this came on, ordered three grains of quinine every hour. He took only six grains when an exacerbation of fever took place, with congestion of liver and stomach. *Prescription.*—℞. Hydrarg. chlor. mit. gr. viii., pulv. ipecac. gr. iv. M. Div. in chart. 4.—S., one every two hours. He took all the powders, which acted gently on the bowels and produced a more perfect remission. Ordered five grains each of quinine and rhubarb, and repeat it in an hour. Soon after the last dose was exhibited, reaction again came on violently, attended with great heat of the skin, thirst, dry tongue, and pulse somewhat tense and irritable, but with little force or fulness. By the use of febrifuge medicines this condition yielded to a partial remission, and I ordered eight grains of quinine, to be repeated in an hour. At my next visit I found my patient without fever, with a cool and moist skin, small, quick and thready pulse, tremulous tongue, and rather torpid intellect. These symptoms soon subsided, and he had a rapid convalescence.

*Case* 3. Attended Mrs. M. æt. about 35, in consultation with the late Dr. John Ker; had been sick about ten days with an obstinate, but not very violent attack of remittent fever; she had previously taken quinine in small doses, been purged, blistered, taken mercury, *&c.* We ordered quinine in ten grain doses, at intervals of two hours; she took twenty grains when her fever rose, having previously to this complained of noises in her ears &c., from the use of the medicine. During the next remission she took thirty grains of quinine in three doses. That night we found our patient exhibiting such symptoms of prostration as obliged us to use strong stimulants for her relief. Her pulse was small, thready and weak, skin cool and clammy, much nausea, and mind torpid. After these were relieved, she began to recover slowly, though for some days she had occasional returns of fever.

*Case* 4. Attended George, about 8 years old, in consultation with Dr. Bagwell of this county. The case was a very severe remittent fever, accompanied by violent congestion of the stomach. He had been previously purged, blistered, &c., and as soon as a partial remission occurred we recommenced giving quinine in five grain doses, repeated every two hours. This was continued during two remissions without any appreciable effect, when Dr. B., who remained with the patient, increased the dose and shortened the interval. The quantity taken in three days is not accurately known, but I should think was not much less than two scruples. As soon as the constitutional effects of the medicine were induced, the disease yielded; but dilatation of the pupil, insensibility to light, and almost total loss of vision followed. The amaurotic condition slowly improved without remedies, and he is now perfectly restored.

I am informed by a physician of this place, that a case similar to some of the above occurred in his family in the course of last fall. A young lady was so prostrated by taking about 20 grains of quinine in two doses, as to require artificial heat to the surface and strong stimulants to restore her. The symptoms were similar to those of the three first cases recorded above.

*Eastville, Northampton, Co.* (*Va.*) *Feb.* 1847.

---

To the Editor of the Medical Examiner.

I have recently observed the evolution of electricity, under circumstances with which I do not know that it has been heretofore noticed.

Standing before a brisk coal fire, and under a large glass chandelier furnished with gas, I was, with other persons in the room, making remarks upon the latter. In the course of these, I touched a tube leading to one of the burners, when there ensued a spark audible to all in the room, and distinctly felt by myself. This was repeated several times by myself as well as the other persons, and many sparks were elicited.

At first I thought that there might be some connexion between the electricity and the combustion of gas, but a moment's reflection convinced me, that the metallic connexion of the burner with the ground rendered this impossible.

It has since occurred to me, that the electricity was generated upon my own person by the friction of my clothes, and that being insulated by the carpet upon which I was standing, I was in the category of the electrical cylinder with the insutedla cushion, which (i. e., the cushion) gives off negative electricity. I had not however the means at hand for testing this.

If this be not the true explanation of the phenomenon, perhaps yourself or some one of your correspondents may be able to furnish it.

*March,* 1847. A. W.

---

To the Editor of the Medical Examiner.

Dear Dr.—In my communication in the last No. of the Med. Examiner, on Wounds from Fire-arms without Ball, the 2d Experiment is thus reported: "Distance six inches, parts covered as before, clothes lacerated, wad lodged one inch and a half below the surface."

This is a material error; it should read, *one half inch* below the surface; and as the experiment may possibly be referred to in future medico-legal inquiries and criminal prosecutions, thou wilt oblige me by giving this correction in the next number of the Examiner.

Respectfully and truly, thy friend,

Paul Swift.

*Philadelphia,* 3d mo. 11th, 1847.

